# Effectiveness of Nemolizumab for Refractory Pruritus in a Pediatric Patient With Atopic Dermatitis After Biliary Atresia Surgery

**DOI:** 10.7759/cureus.83810

**Published:** 2025-05-09

**Authors:** Seiya Masukane, Takeshi Taketani

**Affiliations:** 1 Department of Pediatrics, Shimane University Faculty of Medicine, Izumo, JPN

**Keywords:** atopic dermatitis, biliary atresia, cholestatic pruritus, interleukin-31, nemolizumab, pediatric dermatology

## Abstract

Chronic pruritus following biliary atresia (BA) surgery significantly impairs quality of life, and conventional treatments, including ursodeoxycholic acid, nalfurafine, and antihistamines, often provide insufficient relief. We report a seven-year-old girl with persistent pruritus despite multiple treatments for both cholestatic pruritus and atopic dermatitis (AD). She exhibited severe itching, sleep disturbances, and irritability, refractory to topical corticosteroids, tacrolimus, Janus kinase inhibitors, and systemic therapies. Laboratory findings revealed mild liver dysfunction and elevated total immunoglobulin E, suggesting an allergic predisposition. Given the lack of response to standard therapies, nemolizumab, an interleukin-31 (IL-31) receptor antagonist, was administered. Within one month, pruritus dramatically improved, with her Eczema Area and Severity Index and Patient-Oriented Eczema Measure scores decreasing from 11.0 and 19 to 1.2 and 0, respectively. Sleep disturbances resolved, allowing discontinuation of multiple medications, and her overall well-being significantly improved. This case highlights the potential role of IL-31 in cholestatic pruritus and suggests that IL-31 blockade with nemolizumab may be an effective therapeutic option for refractory pruritus in pediatric patients with BA, warranting further investigation into its broader clinical applications beyond AD.

## Introduction

Biliary atresia (BA) is a rare but severe neonatal liver disease characterized by progressive obliteration of the bile ducts, resulting in cholestasis, liver fibrosis, and eventually cirrhosis if left untreated. The Kasai portoenterostomy, typically performed within the first three months of life, aims to restore bile flow and delay or prevent the need for liver transplantation. Chronic pruritus is a common and distressing symptom in pediatric patients with BA, even after surgical correction. Previous studies have reported that chronic pruritus affects approximately 40% of long-term survivors of BA who have not undergone liver transplantation after Kasai portoenterostomy. This is particularly evident in those with persistent cholestasis, significantly impairing quality of life [1]. The pathophysiology of cholestatic pruritus is multifactorial, involving bile acid accumulation, dysregulation of the endogenous opioid system, and, more recently, lysophosphatidic acid (LPA) signaling mediated by autotaxin (ATX) [2,3].

Conventional therapies, such as ursodeoxycholic acid, rifampin, bile acid-binding resins, and opioid receptor modulators including nalfurafine, often provide insufficient relief, especially in refractory cases [2]. Despite widespread use, antihistamines are largely ineffective for cholestatic itch.

Recent advances have highlighted the role of interleukin-31 (IL-31), a T-helper type 2 (Th2)-derived cytokine, in mediating pruritus through direct activation of sensory neurons via its receptor complex, IL-31 receptor A (IL-31RA) and oncostatin M receptor [4-6]. IL-31 is also implicated in epidermal thickening, skin barrier dysfunction, and chronic itch, independent of histamine pathways [7]. Furthermore, the IL-31/IL-33 axis may amplify inflammatory responses in cutaneous and allergic diseases [8,9], though its role in hepatobiliary conditions has not been established.

Nemolizumab, a humanized monoclonal antibody targeting IL-31RA, has shown significant efficacy in reducing pruritus and improving skin lesions in atopic dermatitis (AD) and prurigo nodularis [10]. We report a pediatric case of severe pruritus following BA surgery, refractory to conventional treatments, that was successfully managed with nemolizumab administered for comorbid AD. This case raises the possibility that IL-31 may also contribute to cholestatic pruritus and highlights the therapeutic potential of IL-31 blockade beyond AD.

## Case presentation

A seven-year-old girl diagnosed with BA during infancy underwent Kasai portoenterostomy at two months of age. Although ursodeoxycholic acid was administered for persistent cholestasis, the itching did not improve, and the eczematous lesions progressively worsened. Moreover, AD was diagnosed at three years of age using the U.K. Working Party criteria [11]. The patient was subsequently treated as follows:

AD treatments include antihistamines (olopatadine, fexofenadine, and others), topical corticosteroids (class II-IV), emollients, topical Janus Kinase inhibitors (delgocitinib), and topical tacrolimus ointment, all applied twice daily.

Cholestatic pruritus treatments consisted of ursodeoxycholic acid (100 mg three times daily) and nalfurafine (2.5 μg once daily).

Management of neurodevelopmental symptoms included risperidone (0.5 mg twice daily), melatonin (1 mg once daily), and multiple herbal medicines (Kanbaku Daiso-to, Yokukansan, and Sai-ko-kei-shi-to at 0.1 g/kg twice daily, respectively). The patient was suspected to have a neurodevelopmental disorder characterized by irritability and restlessness, for which the aforementioned medications were prescribed. However, pruritus and sleep disturbances demonstrated little improvement.

Despite continued symptomatic treatment, pruritus persisted - noticeably impairing sleep, as reported by the caregiver and reflected in Patient-Oriented Eczema Measure (POEM) scores - and interfered with daily activities. She exhibited constant irritability and had difficulty concentrating on her studies, further affecting her quality of life.

At the time of evaluation, laboratory tests revealed mild liver dysfunction, with elevated levels of bilirubin, transaminases, alkaline phosphatase, and gamma-glutamyl transferase. Total immunoglobulin E (IgE) was markedly increased, and dermatophagoides pteronyssinus-specific IgE was strongly positive, suggesting an allergic predisposition.

In addition, given the clinical suspicion of hypohidrosis and a sibling diagnosed with citrin deficiency, genetic testing was performed for Fabry disease and citrin deficiency. Fabry disease was excluded. A heterozygous pathogenic variant in SLC25A13 was identified but was not considered causative. Although progressive familial intrahepatic cholestasis (PFIC) was considered in the differential diagnosis, the persistently elevated gamma-glutamyl transferase level, absence of hepatosplenomegaly, and lack of growth retardation made PFIC unlikely. Genetic testing or liver biopsy for PFIC was not performed.

Historical records showed that liver function parameters had remained within a moderately elevated range over time. These findings are summarized in Table 1, which presents representative laboratory values prior to nemolizumab administration and their historical fluctuations.

**Table 1:** Representative laboratory values before and after nemolizumab administration with historical trends Laboratory findings at the time of evaluation (before nemolizumab) and historical ranges where available. Most liver enzymes, including AST, ALT, ALP, and γ-GTP, were mildly elevated. IgE levels were significantly increased, indicating allergic predisposition. AST, aspartate aminotransferase; ALT, alanine aminotransferase; ALP, alkaline phosphatase; γ-GTP, gamma-glutamyl transferase; IgE, immunoglobulin E; TARC, thymus and activation-regulated chemokine.

Parameter	Historical Range	Baseline (Before Nemolizumab)	At one month after Nemolizumab	At four months after Nemolizumab
Total Bilirubin (mg/dL)	0.6–1.7	1.3	1	1
Direct Bilirubin (mg/dL)	0.2-1.1	0.3	0.3	0.2
AST (U/L)	88-201	193	203	216
ALT (U/L)	84–265	227	241	301
ALP (U/L)	471–645	552	669	648
γ-GTP (U/L)	176–244	176	195	305
Total IgE (IU/mL)	—	713	969	797
D. pteronyssinus-specific IgE (UA/mL)	—	141.28 (Class 6)	—	—
TARC（pg/mL）	—	312.6	461	334

**Figure 1:**
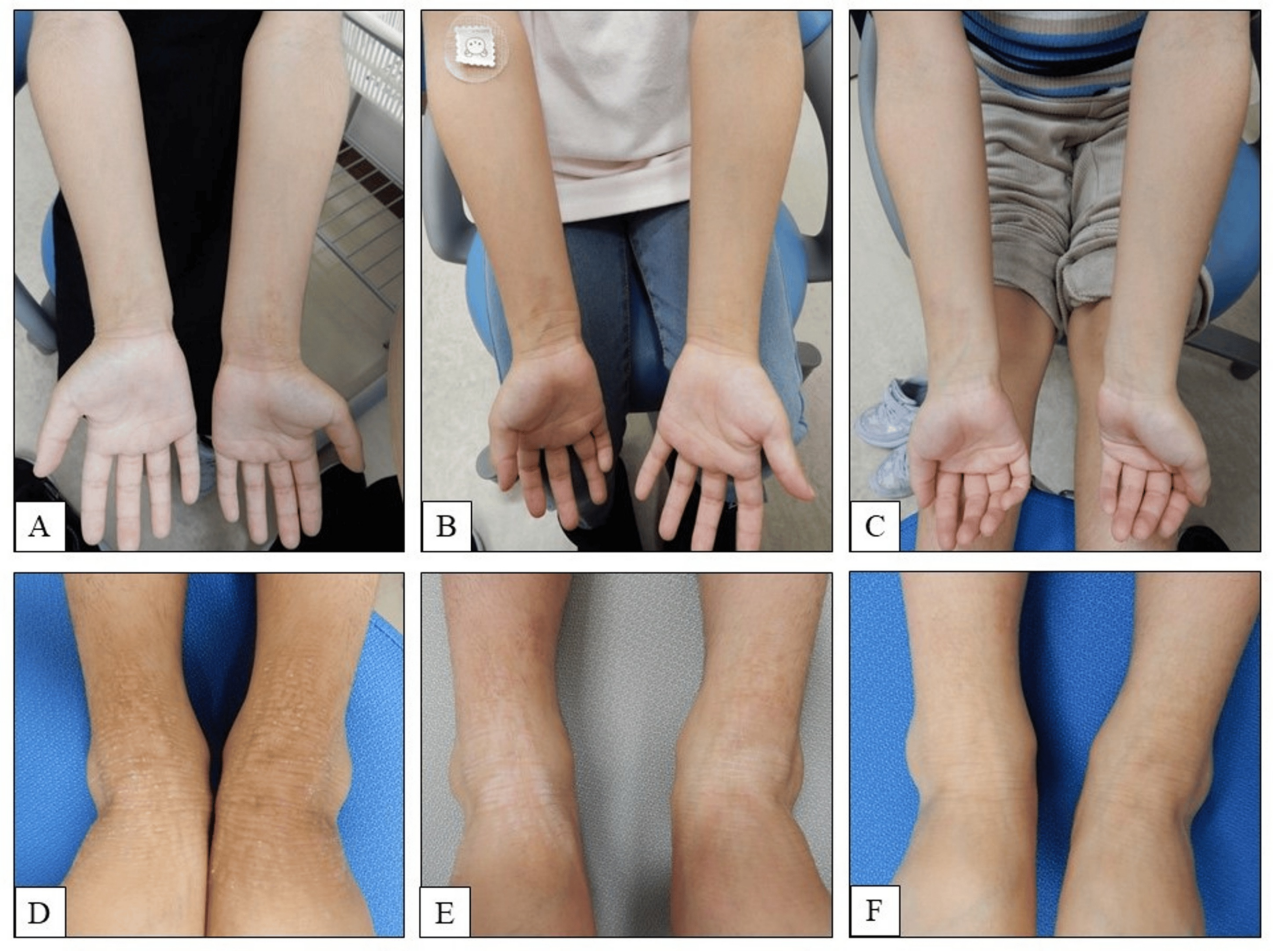
Skin findings before and after nemolizumab treatment. Forearm: before treatment (A), one month post-treatment (B), and two months post-treatment (C). Ankle: before treatment (D), one month post-treatment (E), and two months post-treatment (F). The images demonstrate marked improvement in skin lesions associated with atopic dermatitis (AD). Notably, scratch marks and lichenified plaques on the flexural surfaces of the wrists and ankles showed substantial resolution following treatment. To ensure patient anonymity in accordance with journal policy, moles and other identifiable skin markings were digitally obscured using professional image processing software (Microsoft Photos).

At seven years of age, her Eczema Area and Severity Index (EASI) score was 11.0, and her POEM score was 19, with severe lichenification of the forearms and ankles (Figures 1A, 1D). Despite ongoing therapy, pruritus remained severe. The patient-rated itch severity score (ranging from 0 [none] to 4 [severe]) was 3, indicating moderate to severe itching.

**Figure 2:**
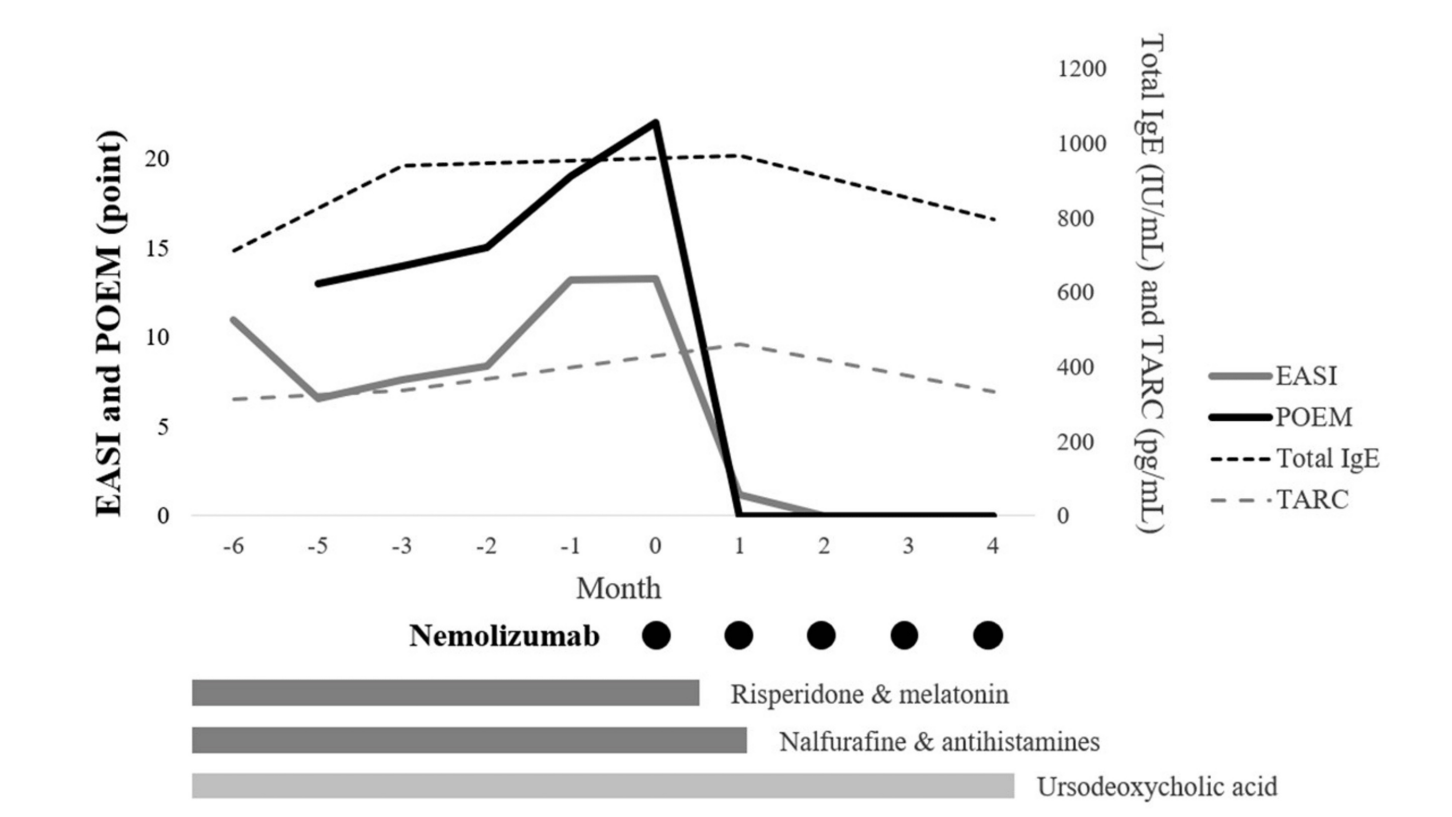
Changes in eczema area and severity index (EASI), patient-oriented eczema measure (POEM), total immunoglobulin E (IgE), thymus and activation-regulated chemokine (TARC) scores before and after nemolizumab treatment. A sharp decrease in EASI and POEM scores is observed following the initiation of nemolizumab. Black dots indicate the timing of subcutaneous nemolizumab (30 mg) administrations, given at four-week intervals. Risperidone, melatonin, nalfurafine, and antihistamines were discontinued after the first month in response to marked improvement in symptoms.

Additionally, nemolizumab (30 mg/kg, subcutaneous) was administered. After one month, pruritus markedly decreased, with her EASI and POEM scores dropping to 1.2 and 0, respectively (Figures 1B, 1E). The itch severity score also improved to 0, indicating complete resolution of subjective itching. Sleep disturbances also resolved, allowing the discontinuation of nalfurafine, antihistamines, risperidone, melatonin, and herbal medicines (Figures 1C, 1F, 2). Her mother subjectively reported an improvement in her overall well-being, evidenced by increased smiling and reduced fatigue. No formal or validated sleep assessment tools were used in this case. She received subcutaneous injections of nemolizumab at a dose of 30 mg every four weeks, in accordance with the approved pediatric regimen for AD. A total of five doses were administered over a four-month period. No adverse events, including injection site swelling or erythema, were observed during or after nemolizumab administration. At present, her pruritus is well controlled with ursodeoxycholic acid and nemolizumab.

## Discussion

Pruritus in BA is often attributed to bile acid accumulation, opioid system dysregulation, and the ATX-LA pathway [2,3]. Although IL-31 has been associated with chronic inflammatory conditions [4,7], the precise role of this cytokine in cholestatic pruritus remains unclear. IL-31 has been associated with various inflammatory and immune-mediated diseases, and its interplay with IL-33 has been suggested to amplify pruritus-related pathways [8,9]. Further studies are needed to determine whether IL-31 contributes directly to cholestatic pruritus.

However, in this case, the substantial and sustained improvement following IL-31 inhibition strongly suggests its involvement in pruritus. Bile acid-modulating therapies, including ursodeoxycholic acid and nalfurafine, did not provide sufficient relief. In contrast, IL-31 inhibition resulted in immediate and sustainable improvement. Notably, the resolution of sleep disturbances enabled the discontinuation of risperidone, melatonin, and herbal medicines, further demonstrating the clinical impact of IL-31 blockade beyond pruritus relief.

Differential diagnoses for cholestatic pruritus, including PFIC, were considered. However, the absence of persistent or progressive elevation in cholestatic liver enzymes and the lack of severe liver dysfunction over time rendered PFIC less likely, and a liver biopsy was not performed. Given the previous clinical suspicion of hypohidrosis and a family history of citrin deficiency, genetic testing was conducted. She was found to be heterozygous for a pathogenic variant in SLC25A13 (associated with citrin deficiency), whereas no pathogenic mutations associated with Fabry disease were identified.

Although nemolizumab was administered under the indication of AD, several clinical features suggested that pruritus in this patient was not solely attributable to AD. First, the pruritus was persistent, severe, and refractory to multiple standard treatments for AD and cholestatic pruritus. Second, the patient had longstanding laboratory abnormalities, including elevated bilirubin, transaminases, and cholestatic enzymes, reflecting chronic liver dysfunction and ongoing cholestasis. Third, the clinical improvement extended beyond cutaneous symptoms to include enhanced sleep, reduced irritability, and discontinuation of neuropsychiatric and sedative medications. These findings indicate that IL-31 blockade may have alleviated pruritus mediated by underlying hepatobiliary dysfunction, in addition to its known effects in AD. Notably, this improvement in pruritus occurred despite only minor fluctuations in liver function parameters, including bilirubin, transaminases, and cholestatic enzymes. This finding supports the hypothesis that IL-31 inhibition exerts its antipruritic effect primarily through modulation of pruriceptive sensory pathways, rather than via direct amelioration of hepatic dysfunction. Consistent with this hypothesis, post-treatment levels of thymus and activation-regulated chemokine and total IgE did not show a consistent decline over time, despite marked clinical improvement. This dissociation further supports the notion that nemolizumab’s therapeutic effect may be mediated predominantly through neural modulation rather than suppression of Th2-driven inflammation.

Nemolizumab has been demonstrated to rapidly reduce pruritus in AD by blocking IL-31RA and disrupting pruriceptive signaling [5,10]. The dramatic response observed in this patient reinforces the importance of IL-31 in chronic pruritus. These findings suggest that IL-31 inhibition may be an effective therapeutic approach for intractable pruritus in AD and cholestatic conditions; however, its precise role in cholestatic pruritus remains unclear. The effectiveness of IL-31 blockade in non-AD-related pruritus, including hepatobiliary disorders, warrants further investigation.

Nevertheless, alternative explanations for the observed clinical improvement must be considered. The natural course of BA can involve fluctuations in liver function and pruritus severity, making spontaneous remission a possible contributor. Moreover, a delayed therapeutic effect of long-term ursodeoxycholic acid administration may have played a role. Other mechanisms, such as potential off-target effects of nemolizumab, also remain unexplored. These possibilities represent key limitations of this case report and highlight the need for controlled studies to elucidate the specific mechanisms underlying the observed effects.

Although the precise mechanisms by which IL-31 blockade alleviates cholestatic pruritus are not fully understood, it is important to acknowledge the established roles of bile acid accumulation, opioid receptor dysregulation, and the ATX-LPA axis in the pathogenesis of cholestatic pruritus. Whether IL-31 signaling interacts with these pathways remains unknown. Future research should investigate whether IL-31 contributes to cholestatic pruritus through direct or indirect modulation of these mechanisms, including any potential overlap with the ATX-LPA axis.

## Conclusions

This case represents the first known pediatric instance of severe pruritus following BA surgery that was successfully managed with nemolizumab administered for comorbid AD. The patient experienced rapid and sustained clinical improvement, including resolution of sleep disturbance, reduced irritability, and discontinuation of multiple medications, suggesting a previously underappreciated role of IL-31 in cholestatic pruritus. Given the limited efficacy of conventional antipruritic therapies in such settings, IL-31 receptor blockade may offer a novel and effective therapeutic approach. This case highlights the need for a broader investigation into cytokine-mediated mechanisms of pruritus in pediatric cholestatic diseases and supports the potential utility of targeted biologics in this population.
